# Emergency department redirection: necessary inconvenience or unacceptable patient experience?

**DOI:** 10.1038/s41390-026-04959-9

**Published:** 2026-04-15

**Authors:** Damian Roland

**Affiliations:** 1 SAPPHIRE Group, Population Health Sciences, Leicester University, Leicester, UK; 2 Paediatric Emergency Medicine Leicester Academic (PEMLA) Group, Children’s Emergency Department, Leicester Royal Infirmary, Leicester, UK

Emergency departments (EDs) manage uncertainty, and in pediatrics, uncertainty is amplified by variable developmental physiology, limited verbal communication, and the emotional weight of caregiver concern. Yet health systems are increasingly asking ED to do something counterintuitive: not simply manage risk, but redistribute it. Redirection from the pediatric ED to community care is one such strategy. It aims to improve flow, better allocate resource and is often a more appropriate patient journey. But it also forces us to confront a fundamental question: who decides that a child does not need to be here?

Qureshi et al.^1^ present a qualitative study exploring how healthcare professionals and health system leaders conceptualize eligibility for redirection from the pediatric ED. Rather than proposing prescriptive criteria, the authors deliberately step back to examine the values and judgments that underpin redirection decisions. Too often, operational algorithms are built before the professional and ethical architecture supporting them has been adequately examined.

Through interviews with pediatric ED clinicians, community physicians, and healthcare leaders, the authors identify three guiding principles to structure decisions about redirection: the impact of the presenting condition on the patient and caregivers, medical complexity, and process considerations. These principles move the debate beyond simple triage-based categorization and toward a more holistic, context-aware approach.

The first principle includes both objective and subjective dimensions. Children considered for redirection must obviously appear physiologically well, with vital signs appropriate for age. However, participants also highlighted that they recognized the influence of caregiver distress and capacity. Caregivers not only attend the ED solely because a symptom exists but also because the situation exceeds their perceived ability to manage safely at home. This is a challenge as there is a spectrum from a first-time parent concerned about a solitary vomit in an infant, to a very experienced mother of 5 children who knows there is something wrong with their (currently) well appearing child, but can’t encapsulate it. Caregiver anxiety, uncertainty, health literacy, and experience become clinically relevant variables. Redirection decisions that ignore these elements risk being technically safe yet practically flawed.

The second principle, medical complexity, extends beyond acuity. Low-acuity, self-limiting conditions such as minor injuries or self-limiting viral illness were commonly viewed as appropriate for potential redirection. In contrast, babies, technology-dependent children, and those likely to require ED-specific diagnostics or interventions were considered less suitable. Importantly, complexity also encompasses system-level factors, such as referral pathway and geographic feasibility. A child referred by another healthcare professional technically represents a different risk profile from a self-referred, otherwise healthy child. However, that risk may be no different from the whole ED cohort if the referrer is inexperienced, hesitant about holding risk in the community, or succumbing to caregiver pressure for definitive management. This broader concept of complexity highlights that redirection is not merely a clinical decision but a function of health system performance.

The third principle, process considerations, addresses how redirection should occur. Participants emphasized that eligibility is dynamic rather than fixed at triage, i.e., a child who initially appears unwell may improve after simple measures, and caregiver concern may diminish with explanation and time. However, determining this, by definition, requires time in the department. To release resource, redirection needs to occur very early in a patient pathway; otherwise, you are creating financial implications for two healthcare providers rather than one. It was also felt that redirection should be offered, not imposed. To preserve autonomy and avoid embedding bias, redirection should be a collaborative pathway rather than a gatekeeping mechanism, particularly for families experiencing structural marginalization.

This latter point highlights the pragmatic challenge of redirection. Principles are clearly necessary, but with no minimum standards or thresholds, redirection affords no benefit to the overcrowded ED. For example, at what age does caution around infancy become exclusion? How should “*low probability of requiring ED intervention*” be operationalized? When does caregiver distress appropriately preclude redirection, and is it possible to refuse to be redirected? There is an uncomfortable truth. If every minor injury, mild viral illness, and stable chronic concern is managed within the ED, families will continue to attend for these conditions. Patterns of attendance are shaped by experience. If the ED consistently provides reassurance, diagnostics, and rapid access, it becomes the default.

Redirection, implemented transparently and safely, can signal that high-quality pediatric care exists beyond hospital walls. But it is a poor experience for families who ultimately make two journeys instead of one, and for a small group of patients, the prospect of being returned to the ED for definitive care is inevitable because disease processes have emerged such that specialist care is necessary. Shared decision-making is presented as the ethical anchor, but this requires time, communication resources, and institutional support. This can be at odds with the pressure placed on ED triage systems to sift and sort patients efficiently and effectively.

As with all qualitative work, context matters. This was a single-site study conducted within a specific Canadian healthcare environment. The transferability of these guiding principles to health systems with different primary care accessibility, funding models, or medico-legal climates remains uncertain. However, in this author’s experience, the problems highlighted in this paper are applicable worldwide. It is critical that caregiver and patient perspectives are included in future phases of this type of work. And without prospective safety evaluation and transparent reporting of outcomes, including re-attendance, adverse events, and caregiver satisfaction, redirection risks remain conceptually attractive but operationally fragile.

The task, therefore, is not whether redirection should occur, but how it can be embedded safely, equitably, and transparently within pediatric emergency systems. Qureshi and colleagues provide a thoughtful conceptual scaffold for this next phase of work. Redirection should never become a euphemism for rationing. Nor should it be abandoned because it is difficult. Pediatric emergency care has always required the careful balancing of vigilance and proportionality. Extending that balance beyond the ED walls, while maintaining trust, may be one of the defining service design challenges of the coming decade.
